# Sunitinib-Induced Elevation of Mean Corpuscular Volume (MCV)—Exploring Its Possible Clinical Relevance in Cancer Patients

**DOI:** 10.3390/curroncol29060330

**Published:** 2022-06-07

**Authors:** Michal Rihacek, Iveta Selingerova, Ivo Kocak, Ilona Kocakova, Eva Rihackova, Dalibor Valik, Jaroslav Sterba

**Affiliations:** 1 Department of Pediatric Oncology, Faculty of Medicine, University Hospital Brno and Masaryk University, Cernopolni 9, 613 00 Brno, Czech Republic; rihacek.michal@fnbrno.cz (M.R.); valik.dalibor@fnbrno.cz (D.V.); sterba.jaroslav@fnbrno.cz (J.S.); 2 Department of Laboratory Medicine, Masaryk Memorial Cancer Institute, Zluty kopec 7, 656 53 Brno, Czech Republic; 3 Department of Laboratory Medicine, University Hospital Brno, Jihlavska 20, 625 00 Brno, Czech Republic; 4 Department of Biochemistry, Faculty of Medicine, Masaryk University, Kamenice 5, 625 00 Brno, Czech Republic; 5 Department of Pharmacology, Faculty of Medicine, Masaryk University, Kamenice 5, 625 00 Brno, Czech Republic; 6 Department of Comprehensive Cancer Care, Masaryk Memorial Cancer Institute, Žlutý kopec 7, 656 53 Brno, Czech Republic; kocak@mou.cz (I.K.); kocakova@mou.cz (I.K.); 7 Department of Comprehensive Cancer Care, Faculty of Medicine, Masaryk University, Žlutý kopec 7, 625 00 Brno, Czech Republic; 8 Department of Internal Cardiology Medicine, Faculty of Medicine, University Hospital Brno and Masaryk University, Jihlavska 20, 625 00 Brno, Czech Republic; rihackova.eva@fnbrno.cz; 9 Department of Laboratory Methods, Faculty of Medicine, Masaryk University, Kamenice 5, 625 00 Brno, Czech Republic; 10 The International Clinical Research Centre of St. Anne’s University Hospital in Brno, Pekarska 53, 656 91 Brno, Czech Republic

**Keywords:** sunitinib, mean corpuscular volume, MCV, toxicity

## Abstract

Sunitinib is a broad-spectrum multitargeted tyrosine kinase inhibitor mainly used as second-line therapy for non-resectable gastrointestinal stromal or first-line treatment option of metastatic renal cell carcinoma (mRCC), and as an “off-label” option in pediatric oncology. It has been previously reported that sunitinib elevates the mean corpuscular volume of erythrocytes (MCV) in treated subjects. The aim of this study was to assess time-dependent changes of this effect and evaluate its possible clinical relevance. In this study, 179 adult and 21 pediatric patients with solid tumors treated with sunitinib were retrospectively analyzed. The laboratory and treatment-related data were collected for each treatment period. The regression model with a broken-line relationship was used to fit time dependence of the MCV. In the adult group, the MCV was increasing during the first 21.6 weeks (median) of treatment in a median level of 99.8 fL, where it stabilized. MCV increase was faster in the patients who suffered from treatment-related adverse events (21.3 vs. 24.6 weeks, *p* = 0.010). In the pediatric cohort, the MCV dynamics were similar to adults. In conclusion, MCV changes during sunitinib treatment in pediatric and adult patients may be of clinical utility in monitoring sunitinib treatment course.

## 1. Introduction

Sunitinib is an oral anticancer drug from a class of tyrosine kinase inhibitors that target multiple intracellular molecular signaling pathways, including vascular endothelial growth factor receptors (VEGFR) pathway, platelet-derived growth factor receptors (PDGFR) pathway, stem cell growth receptor (c-KIT) pathways, FMS-like tyrosine kinase 3 (FLT3), colony-stimulating factor 1 receptor (CSF-1R) pathway, and “rearranged during transfection” (RET) receptor pathway [1]. Since 2006, it has been approved for the treatment of metastatic renal cell carcinoma (mRCC) [2,3] and gastrointestinal stromal tumor (GIST) [4,5].

A recent meta-analysis on therapeutic alternatives to first-line sunitinib monotherapy in mRCC patients demonstrated the benefit of using combined immune therapy with checkpoint inhibitors [6]. A careful approach should be taken in patients with dysregulated immune activation such as pre-existing autoimmune diseases or hematopoietic/solid organ transplant or compromised immune function (long-term immunosuppression, chronic viral infections), and/or significant medical co-morbidities (organ dysfunction, elderly or frail patients, metastatic brain disease) [7]. However, these patients and possibly those with favorable risk may still benefit from sunitinib monotherapy [8]. Furthermore, the benefit of a selective sequential treatment strategy has been proposed [7].

Although studies on pediatric patients have been conducted mostly with GIST and RCC in a limited number of pediatric patients [9,10,11], it remains an “off-label” option for a “targeted” pharmacotherapy approach in high-risk pediatric CNS tumors and other pediatric malignancies that have a specific molecular microenvironment favoring the use of sunitinib in the therapeutic protocol [12].

Sunitinib and its main metabolite N-desethyl-sunitinib are indolinone derivatives with lipophilic properties, high distribution volume (V_d_ = 2030 l and 3080 l, respectively) and half-life of 40–60 h and 80–110 h, respectively [13]. Therefore, approximately 10–14 days are required for reaching steady-state concentration in most patients, including those with renal insufficiency, as both the drug and its metabolite are mainly excreted in feces [14,15]. A therapeutic drug monitoring data summary from a recent review study proposed cumulative concentration of sunitinib and N-desethyl-sunitinib of 50–100 ng/mL as an appropriate target therapeutic window as concentrations over 100 ng/mL may lead to increased toxicity [16]. Nevertheless, no routine rapid diagnostic (STAT-based) method for therapeutic drug monitoring is currently available to guide dosing.

It has been reported that sunitinib and several other tyrosine kinase inhibitors influence the erythrocyte mean corpuscular volume parameter (MCV) [17,18]. Initial considerations about this phenomenon included possible relative folate and cobalamin deficiency [19,20]. However, more recent studies provided data that this effect is related to the inhibitory activity of tyrosine kinase inhibitors towards c-KIT, which is extensively expressed by progenitor cells in the bone marrow [21,22,23]. This theory is supported by results of studies where imatinib, sunitinib, and pazopanib (c-KIT inhibitors) treatment is associated with a statistically significant rise in the MCV, whereas sorafenib, erlotinib, and vemurafenib (no c-KIT inhibitory activity) showing no such association [12,17,18,24]. Therefore, we were motivated to conduct a structured retrospective study to assess whether this pharmacotherapy-induced epiphenomenon may be clinically informative.

## 2. Materials and Methods

A retrospective study was performed in adult patients treated with sunitinib (*n* = 179, overall) between 2008 and 2021 at the Masaryk Memorial Cancer Institute (MMCI) and selected according to the following inclusion criteria: application of sunitinib as a single agent in patients not treated with any TK inhibitor for a sufficient “washout” period (at least 6 months, to prevent possible alteration of the MCV baseline). Every sunitinib treatment period separated by a washout period for one patient was considered independently. Patients with an insufficient number of blood collections for the evaluation of selected parameter dynamics and those without sufficient data about the treatment (incomplete patient history data, patients with treatment interruptions due to various clinical reasons) were not included in this study. A cohort of pediatric patients treated with sunitinib in an individual “off-label” regimen at the Department of Pediatric Oncology Hospital Brno for various types of malignancies between years 2012 and 2019 was selected with the same inclusion and exclusion criteria as described for adults. As pediatric patients did not have standardized treatment protocols, the dosing was mostly adjusted according to the age, body mass, treatment tolerance, and clinical toxicity. The main goal was also to assess the dynamics of the MCV as the authors were not aware of any previous report on this topic in pediatric patients.

In adults, the standard treatment cycle of sunitinib was 50 mg/day in a 6-week course: 4 weeks on, 2 weeks off. Some patients had a dose reduction to 37.5 mg a day or 25 mg a day, respectively, due to possible drug-related adverse events. Dosage interruptions and adjustments for toxicity or intolerance were performed according to the manufacturer’s recommendations. Treatment periods, initial and/or adjusted sunitinib doses, were obtained from pharmacy reports. In the pediatric cohort, individual dosing regimens were employed and managed according to body surface area (BSA), age, and treatment tolerance, ranging from 7 to 32 mg/m^2^/day.

Collected laboratory data included retrospective results of patients’ complete blood counts (CBC) with white blood cell differential (WBCD) and basic clinical chemistry tests and anemia-related chemistry parameters (electrolytes, blood urea nitrogen, creatinine, liver function tests, vitamin B12, and folic acid) if available. Hematology parameters (CBC, WBCD) were measured on Sysmex XE 5000 or Sysmex XN 2000 (with verified comparability of the results between both analyzers), whereas clinical chemistry parameters were assayed by the Cobas 6000 c501 chemistry and e411/e601 electrochemiluminescence module (folic acid, vitamin B12). Adverse effects evaluated as treatment-related, and patient treatment outcomes data were collected from clinical reports of outpatient visits or hospital admission and discharge reports.

**Figure 1:**
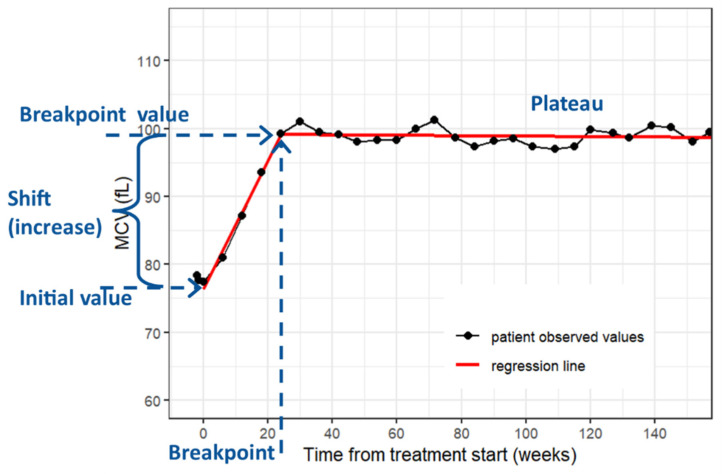
Illustration of statistical modeling of MCV time-courses and description of terminology.

Patient and treatment period characteristics were described using standard summary statistics, i.e., median and interquartile range (IQR) for continuous variables and frequencies and proportions for categorical variables. Based on exploratory analysis, MCV time-courses for individual treatment periods were fitted using a segmented linear regression model with one breakpoint (Figure 1) [25]. The slope of the regression line in the first segment represents an MCV increase per week. The sunitinib treatment periods were further divided into groups depending on whether they were considered sufficient for statistical analysis of full MCV rise pattern. Treatment periods with at least three MCV measurements and at least four weeks of ongoing treatment after breakpoint were labeled as a full period group (*n* = 144), and the others were labeled as a censored period group (*n* = 56). The common regression was provided using a segmented linear mixed regression model with one breakpoint. All statistical analyses were performed employing the R version 4.1.3 and a common significance level of 0.05 [26].

## 3. Results

The analyzed adult group included 179 retrospectively selected patients. Patient characteristics are reported in Table 1. A total of evaluated sunitinib treatment periods were 200, wherein 16 patients (9%) had two or more periods.

**Figure 2:**
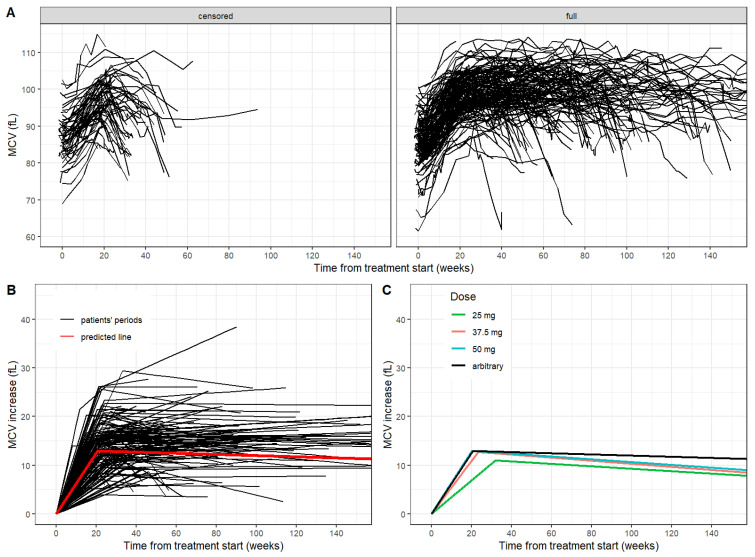
(**A**) Time-courses of the MCV according to censored and full period groups. (**B**) Fit regression models with broken-line relationships for patient periods in full period groups (black) and common predicted line (red). (**C**) Dose-related predicted lines for the full period group.

The median initial value of the MCV at the start of the treatment period was 85.4 fL. The median treatment duration was 77.4 and 28.4 weeks in groups of full and censored periods, respectively. During treatment discontinuation, the patient’s clinical condition was most frequently progression of the disease (PD) in 146 periods (73%). A total of 116 periods (59%) were dose-adjusted. Other characteristics of treatment periods are summarized in Table 2. The time-courses of the MCV in both groups are shown in Figure 2A.

The median increase of the MCV after the treatment start was 0.67 fL and 0.58 fL per week in groups of full and censored periods, respectively. In the group of full periods, a specific pattern of the MCV was observed. After the initial increase of the MCV, the MCV level was stabilized (plateau) with continuing treatment and declined when the treatment was ceased. The breakpoint of MCV increase showed a median of 21.6 weeks (IQR 18.8–25.7). The MCV value in the breakpoint-time had a median of 99.8 fL. The median MCV shift was 14.4 fL. The common regression model for the full period group yielded estimates of the initial increase of the MCV as 0.63 fL per week, the breakpoint of the MCV increase as 20.6 weeks (Figure 2B).

To compare the possible impact of treatment adjustment, full periods where patients received a single dose of 25 mg (6 periods), 37.5 mg (19 periods) and 50 mg (25 periods), respectively, without any dose adjustment, were selected. Subsequently, the dose-related effect was evaluated in the full periods group with a single dose of 25 mg, 37.5 mg and 50 mg, respectively, throughout the whole treatment period. We observed a statistically significant difference in MCV breakpoint value and MCV increase per week after treatment start between dose groups (*p* = 0.006, *p* = 0.019). However, initial, final and shift values of MCV were comparable between those groups (Table 3, Figure 2C).

The possible prognostic impact of observed MCV changes was evaluated in the context of toxicity and treatment outcome. Documented treatment of adverse events were sorted according to their origin to related organ system (cardiovascular, dermatological, hematotoxicity, etc.). The most common adverse events were gastrointestinal adverse events (118 periods, 41%) and hematotoxicity (64 periods, 32%). No adverse events were reported in 42 periods (21%). A statistically significant difference between periods with any reported adverse event and with no adverse events was observed in MCV increase per week value (median 0.69 vs. 0.53 fL, *p* = 0.020) and breakpoint value (21.3 vs. 24.6 weeks, *p* = 0.010), suggesting a faster MCV increase rate in those who suffer from treatment-related adverse events.

With regards to treatment outcome and the possible utility of MCV as an independent predictor of survival, we divided the group of full periods according to the disease state during treatment discontinuation from any possible clinical reason. Two groups were further evaluated—progression of the disease (PD) and stable disease/partial remission (PR + SD). As expected, no complete remission (CR) in an adult patient was observed. No statistically significant difference was observed between these two groups (Table 4).

The analyzed pediatric group included 21 retrospectively selected patients with various clinical conditions (Table 5).

**Figure 3:**
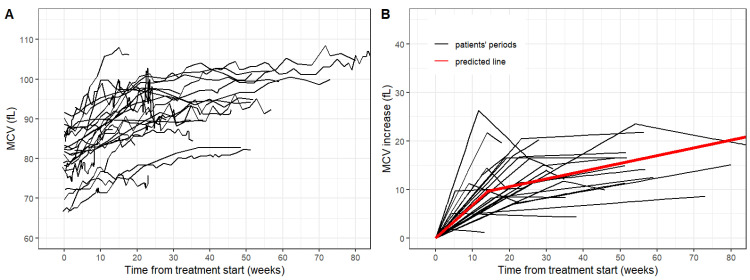
(**A**) Time-courses of MCV. (**B**) Fit regression models with broken-line relationships (black) and common predicted line (red) for pediatric cohort.

The median initial value of the MCV at the start of the treatment period was 80.2 fL. The median treatment duration was 39.0 weeks. During treatment discontinuation, a patient’s clinical condition was most frequently progression of the disease (PD) in 11 periods (46%). More patients with SD and CR were observed compared to adult patients, but these patients often received concomitant conventional chemotherapy in various treatment protocols. Other characteristics of treatment periods are summarized in Table 5. The time-courses of the MCV in both groups are shown in Figure 3.

The median increase of the MCV after the treatment start was 0.69 fL per week. Statistical modeling of MCV time-courses yielded a similar pattern of the MCV as observed in the adult group. The breakpoint of MCV increase showed a median of 20 weeks (IQR 12.5–23.7) similar to that of the adult group. The median of MCV shift was 12.4 fL.

## 4. Discussion

Macrocytosis results from either abnormal development of a cell or its membrane composition or increased reticulocyte count. These events may be present coincidentally. Initial observations published by Gillessen and Billemont [19,27] and supported by another case report by Reed et al. [20] suggested a connection between absolute or relative vitamin B12 deficiency and sunitinib-related macrocytosis. However, these observations were performed on a limited number of patients.

Our results showed that significant elevation of the MCV can be observed in the vast majority of patients receiving sunitinib for a period of at least four cycles, which is in concordance with studies by Price et al. [23], Kloth et al. [17], and Bourlon et al. [22]. Vitamin B12 and/or folic acid deficiency was not observed in most cases in our patients who had results of these parameters available during sunitinib treatment (data not shown). Therefore, we incline to the alternative explanation proposed by Schallier et al. [18] and Galanis et al., [24] that this effect is more likely mediated by sunitinib inhibitory activity on c-KIT signaling that is an important molecular event in hematopoiesis. This is also consistent with available white blood cell differential morphology results in our adult patient cohort during sunitinib treatment not showing any specific abnormalities associated with severe vitamin B12 and/or folic acid deficiency or with severe myelodysplasia (hypersegmentation of neutrophil, Howell-Jolly bodies etc., hyposegmentation of neutrophils). However, more prospective studies need to be conducted to clarify the underlying pathophysiology of this phenomenon that would include regular vitamin B12, folic acid, and iron metabolism parameters together with evaluation of blood smear microscopy for possible abnormalities in blood cell morphology. Concurrent analysis of both TK inhibitors that target c-KIT (e.g., axitinib, pazopanib also used in the treatment of RCC) and those that do not provide clinically relevant data.

Our observations on MCV dynamics in patients treated with sunitinib are also consistent with those by Kloth et al. [17] in a similar number of subjects (179 vs. 213). We calculated regression models of MCV dynamics in our cohort as we assumed that the MCV might have been a valuable parameter for therapeutic monitoring using a surrogate marker. In studies by Kloth et al. and Bourlon et al., a statistically significant positive correlation was shown between macrocytosis and progression-free survival time, the latter further showing that patients with treatment-related hypothyroidism have more favorable outcomes than those with normal thyroid functions [17,22]. A more recent study by Kucharz et al. [28] observed longer progression-free survival in those subjects who reached a MCV >100 fL after three cycles, an observation that we did not find (data not presented). Taken together, we did not observe a statistically significant relation between observed MCV parameters and treatment outcomes.

Regarding treatment toxicity, we observed a very similar array of adverse events to those published in the literature and safety product sheet, with gastrointestinal and hematotoxicity being the most common [29,30]. Interestingly, the MCV increase rate was higher in those subjects suffering from any adverse events than in those that did not have any adverse events reported. Novel approaches emerge in the treatment of RCC including immune therapy or sequential therapies that phase out TK inhibitor monotherapy in RCC patients. It follows that new surrogate biomarkers may be of help in identifying agent-specific toxicities in combination, i.e., sunitinib-containing therapies.

Studies on the pediatric population treated with sunitinib yielded limited results mostly due to the complexity of the treatment context, a limited number of patients, and ethical concerns as well [9,10,11]. Despite that, we were able to collect limited but relevant data demonstrating that the pharmacological mechanism behind this epiphenomenon may not differ between adult and pediatric populations. Of note, sunitinib-induced elevation of the MCV was apparently not affected by concomitant anti-cancer polychemotherapy in pediatric patients.

Our study has the following limitations. This was a retrospective study on a limited number of patients. We did not specifically address the pathophysiology of B12/folate-dependent red blood cell morphology as we did not have retrospective specimens available. The pediatric part of the study had even fewer patients included; therefore, fewer observations were available. Due to the retrospective nature of this study, the respective specimen sampling was not fully standardized with respect to sampling times, adverse events, or clinical toxicity evaluations.

## 5. Conclusions

We describe a phenomenon of MCV elevations occurring in the course of treatment with a multitargeted tyrosine kinase inhibitor sunitinib. This is an apparent drug-related epiphenomenon where the underlying pharmacological mechanism may go through c-KIT signaling pathway inhibition. We present a regression model of this phenomenon for standard dosing that may serve as an additional but perhaps valuable surrogate marker for therapeutic drug monitoring and treatment adherence indicator for pediatric and adult patients. Adult patients that develop any adverse events appear to have a faster MCV increase rate than those reporting no adverse event, hence, suggesting its possible clinical utility as an indicator of therapeutic toxicity.

## Figures and Tables

**Table 1:** Patient characteristics.

Characteristic	*n* = 179
Gender	
Female	44 (25%)
Male	135 (75%)
Age at treatment start (years)	
Median (IQR)	62 (56, 68)
Range	28–81
Diagnosis	
metastatic renal cell carcinoma (mRCC)	161 (91%)
gastrointestinal stromal tumor (GIST)	17 (7.3%)
alveolar sarcoma	1 (0.6%)
Included number of treatment periods	
1	163 (91%)
2	13 (7.3%)
3	2 (1.1%)
5	1 (0.6%)

Abbreviation: IQR, interquartile range.

**Table 2:** Treatment period characteristics and observed parameters of MCV time-courses.

Characteristic (Median, IQR)	Overall *n* = 200	Full *n* = 144	Censored *n* = 56
MCV (fL)			
initial value	85.4 (81.9, 89.2)	85.1 (81.7, 89.0)	86.4 (82.9, 89.3)
breakpoint value	NS	99.8 (96.2, 103.0)	NS
MCV shift	NS	14.4 (11.5, 16.6)	NS
MCV increase per week (fL)	0.65 (0.48, 0.82)	0.67 (0.52, 0.86)	0.58 (0.46, 0.72)
Breakpoint time (weeks)	NS	21.6 (18.8, 25.7)	NS
Treatment duration (weeks)	60.2 (37.9, 96.4)	77.4 (53.6, 113.8)	28.4 (25.8, 37.3)
Patient clinical condition at the moment of treatment discontinuation			
PD	146 (73%)	104 (72%)	42 (75%)
SD	42 (21%)	32 (22%)	10 (18%)
PR	12 (6.0%)	8 (5.6%)	4 (7.1%)

Abbreviations: NS, not specified; MCV, mean corpuscular volume; PD, progressive disease; SD, stable disease; PR, partial response; IQR, interquartile range.

**Table 3:** Observed parameters of MCV time-courses and sunitinib dose-related effect in the group of full periods.

Characteristic (Median, IQR)	25 mg *n* = 6	37.5 mg *n* = 19	50 mg *n* = 25	*p*-Value
MCV (fL)				
initial value	87.1 (84.3, 92.1)	88.4 (85.5, 90.7)	85.2 (83.0, 89.7)	0.294
breakpoint value	102.0 (99.5, 102.4)	101.7 (97.4, 104.1)	98.7 (95.6, 101.2)	0.210
MCV shift	12.8 (11.6, 13.3)	13.0 (10.5, 15.2)	13.0 (11.1, 15.9)	0.874
MCV increase per week (fL)	0.43 (0.39, 0.50)	0.55 (0.41, 0.67)	0.67 (0.55, 0.79)	0.019
Breakpoint time (weeks)	29.1 (24.3, 45.6)	23.0 (21.7, 24.4)	20.1 (16.7, 22.2)	0.006
Treatment duration (weeks)	92.6 (87.5, 102.1)	66.0 (50.4, 93.9)	60.7 (45.1, 74.3)	0.074

Abbreviations: MCV, mean corpuscular volume; IQR, interquartile range.

**Table 4:** Observed parameters of MCV time-courses and relation to patient clinical condition at the moment of treatment discontinuation in the group of full periods.

Characteristic (Median, IQR)	PD *n* = 103	PR + SD *n* = 40	*p*-Value
MCV (fL)			
initial value	85.2 (82.2, 89.3)	85.1 (80.6, 88.4)	0.310
breakpoint value	100.0 (96.2, 103.3)	98.7 (96.3, 102.1)	0.736
MCV shift	14.4 (11.1, 16.5)	14.5 (12.1, 16.7)	0.516
MCV increase per week (fL)	0.68 (0.52, 0.86)	0.66 (0.53, 0.82)	0.867
Breakpoint time (weeks)	21.9 (18.0, 25.7)	21.5 (20.0, 25.4)	0.641
Treatment duration (weeks)	76.7 (51.8, 108.5)	77.6 (60.5, 118.2)	0.471

Abbreviations: MCV, mean corpuscular volume; PD, progressive disease; SD, stable disease; CR, partial response; IQR, interquartile range.

**Table 5:** Patient (left) and treatment period (right) characteristics and observed parameters of MCV time-courses of pediatric cohort.

Patients		Treatment Periods	
Characteristic	*n* = 21	Characteristic (Median, IQR)	*n* = 24
Gender		MCV (fL)	
Female	8 (38%)	initial value	80.2 (76.6,85.2)
Male	13 (62%)	breakpoint value	92.2 (88.0, 98.0)
Age at treatment start (years)		MCV shift	12.4 (8.4, 16.5)
Median (IQR) Range	7.7 (4.5, 13.3) 4 months–22.9 years	MCV increase per week (fL)	0.69 (0.45, 1.07)
		Breakpoint time (weeks)	20.0 (12.5, 23.7)
Diagnosis		Treatment duration (weeks)	39.0 (23.25, 57.6)
carcinoma	1 (4.8%)	Patient clinical condition at the moment of treatment discontinuation	
mesenchymal tumors	5 (24%)		
neuroblastoma and primary		PD	11 (46%)
CNS tumors	3 (14.3%)	SD	5 (21%)
sarcomas	7 (33%)	PR	1 (4.2%)
vascular neoplasia	5 (24%)	CR	7 (29 %)
Included number of treatment periods			
1	18 (86%)		
2	3 (14%)		

Abbreviations: MCV, mean corpuscular volume; PD, progressive disease; SD, stable disease; PR, partial response; CR, complete response; IQR, interquartile range.

## Data Availability

The data presented in this study are available on request from the corresponding author.

## References

[1] Mena A.C., Pulido E.G., Guillén-Ponce C. (2010). Understanding the molecular-based mechanism of action of the tyrosine kinase inhibitor: Sunitinib. Anticancer Drugs.

[2] Poprach A., Büchler T., Lakomy R., Tomasek J. (2014). Targeted therapy of metastatic renal cell carcinoma and treatment sequences: A current view. Onkologie.

[3] Poprach A., Lakomy R., Bortlicek Z., Melichar B., Pavlik T., Slaby O., Vyzula R., Svoboda M., Kiss I., Studentova H. (2016). Efficacy of Sunitinib in Elderly Patients with Metastatic Renal Cell Carcinoma: Data from Real-World Clinical Practice. Drugs Aging.

[4] Kocakova I., Kocak I., Spelda S., Krejci E., Bencsikova B., Jureckova A., Vyzula R., Bortlicek Z., Strenkova J., Brabec P. (2015). Long term experience of patients with unresectable or metastatic KIT positive gastrointestinal stromal tumours. Bratisl. Lek. Listy.

[5] Bencsikova B. (2015). Targeted therapy based treatment of gastrointestinal stromal tumors. Onkologie.

[6] Massari F., Rizzo A., Mollica V., Rosellini M., Marchetti A., Ardizzoni A., Santoni M. (2021). Immune-based combinations for the treatment of metastatic renal cell carcinoma: A meta-analysis of randomised clinical trials. Eur. J. Cancer.

[7] Rossi E., Bersanelli M., Gelibter A.J., Borsellino N., Caserta C., Doni L., Maruzzo M., Mosca A., Pisano C., Verzoni E. (2021). Combination Therapy in Renal Cell Carcinoma: The Best Choice for Every Patient?. Curr. Oncol. Rep..

[8] Rini B.I., Plimack E.R., Stus V., Gafanov R., Hawkins R., Nosov D., Pouliot F., Alekseev B., Soulières D., Melichar B. (2019). Pembrolizumab plus Axitinib versus Sunitinib for Advanced Renal-Cell Carcinoma. N. Engl. J. Med..

[9] Janeway K.A., Albritton K.H., Van Den Abbeele A.D., D’Amato G.Z., Pedrazzoli P., Siena S., Picus J., Butrynski J.E., Schlemmer M., Heinrich M.C. (2009). Sunitinib treatment in pediatric patients with advanced GIST following failure of imatinib. Pediatr. Blood Cancer.

[10] Dubois S.G., Shusterman S., Ingle A.M., Ahern C.H., Reid J.M., Wu B., Baruchel S., Glade-Bender J., Ivy P., Grier H.E. (2011). Phase I and pharmacokinetic study of sunitinib in pediatric patients with refractory solid tumors: A children’s oncology group study. Clin. Cancer Res..

[11] DuBois S.G., Shusterman S., Reid J.M., Ingle A.M., Ahern C.H., Baruchel S., Glade-Bender J., Ivy P., Adamson P.C., Blaney S.M. (2012). Tolerability and pharmacokinetic profile of a sunitinib powder formulation in pediatric patients with refractory solid tumors: A Children’s Oncology Group study. Cancer Chemother. Pharmacol..

[12] Krchniakova M., Skoda J., Neradil J., Chlapek P., Veselska R. (2020). Repurposing Tyrosine Kinase Inhibitors to Overcome Multidrug Resistance in Cancer: A Focus on Transporters and Lysosomal Sequestration. Int. J. Mol. Sci..

[13] Houk B.E., Bello C.L., Kang D., Amantea M. (2009). A population pharmacokinetic meta-analysis of sunitinib malate (SU11248) and its primary metabolite (SU12662) in healthy volunteers and oncology patients. Clin. Cancer Res..

[14] Verschuur A.C., Bajciova V., Mascarenhas L., Khosravan R., Lin X., Ingrosso A., Janeway K.A. (2019). Sunitinib in pediatric patients with advanced gastrointestinal stromal tumor: Results from a phase I/II trial. Cancer Chemother. Pharmacol..

[15] Izzedine H., Etienne-Grimaldi M.C., Renée N., Vignot S., Milano G. (2009). Pharmacokinetics of sunitinib in hemodialysis. Ann. Oncol..

[16] Demlova R., Turjap M., Pes O., Kostolanska K., Jurica J. (2020). Therapeutic Drug Monitoring of Sunitinib in Gastrointestinal Stromal Tumors and Metastatic Renal Cell Carcinoma in Adults—A Review. Ther. Drug Monit..

[17] Kloth J.S.L., Hamberg P., Mendelaar P.A.J., Dulfer R.R., van der Holt B., Eechoute K., Wiemer E.A.C., Kruit W.H.J., Sleijfer S., Mathijssen R.H.J. (2016). Macrocytosis as a potential parameter associated with survival after tyrosine kinase inhibitor treatment. Eur. J. Cancer.

[18] Schallier D., Trullemans F., Fontaine C., Decoster L., De Greve J. (2009). Tyrosine kinase inhibitor-induced macrocytosis. Anticancer Res..

[19] Billemont B., Izzedine H., Rixe O. (2007). Macrocytosis due to treatment with sunitinib. N. Engl. J. Med..

[20] Reed J.P., Chung J., Banerjee N. (2019). A Case of Cobalamin Deficiency and Macrocytic Anemia Secondary to Sunitinib. Cureus.

[21] Rini B.I., Choueiri T.K., Elson P., Khasawneh M.K., Cotta C., Unnithan J., Wood L., Mekhail T., Garcia J., Dreicer R. (2008). Sunitinib-induced macrocytosis in patients with metastatic renal cell carcinoma. Cancer.

[22] Bourlon M.T., Gao D., Trigero S., Clemons J.E., Breaker K., Lam E.T., Flaig T.W. (2016). Clinical significance of sunitinib-associated macrocytosis in metastatic renal cell carcinoma. Cancer Med..

[23] Price J., Shaarbaf R., Wood L. (2010). Sunitinib causes macrocytosis in patients with advanced renal cell carcinoma. Curr. Oncol..

[24] Galanis A., Levis M. (2015). Inhibition of c-Kit by tyrosine kinase inhibitors. Haematologica.

[25] Muggeo V.M. (2003). Estimating regression models with unknown break-points. Stat. Med..

[26] R Core Team R: A Language and Environment for Statistical Computing. https://www.R-project.org/.

[27] Gillessen S., Graf L., Korte W., Cerny T. (2007). Macrocytosis and cobalamin deficiency in patients treated with sunitinib. N. Engl. J. Med..

[28] Kucharz J., Giza A., Dumnicka P., Kuzniewski M., Kusnierz-Cabala B., Bryniarski P., Herman R., Zygulska A.L., Krzemieniecki K. (2016). Macrocytosis during sunitinib treatment predicts progression-free survival in patients with metastatic renal cell carcinoma. Med. Oncol..

[29] Demetri G.D., van Oosterom A.T., Garrett C.R., Blackstein M.E., Shah M.H., Verweij J., McArthur G., Judson I.R., Heinrich M.C., Morgan J.A. (2006). Efficacy and safety of sunitinib in patients with advanced gastrointestinal stromal tumour after failure of imatinib: A randomised controlled trial. Lancet.

[30] Ibrahim E.M., Kazkaz G.A., Abouelkhair K.M., Bayer A.M., Elmasri O.A. (2013). Sunitinib adverse events in metastatic renal cell carcinoma: A meta-analysis. Int. J. Clin. Oncol..

